# Clinical characteristics and a screening tool for VEXAS syndrome: a case-control study from China

**DOI:** 10.1186/s13023-026-04380-9

**Published:** 2026-06-18

**Authors:** Menghui Yao, Min Shen, Chengjin Huang, Yang Jiao, Yue Sha, Xianyong Jiang, Xiaoxi Yang, Jun Feng, Na Xu, Weiguo Zhu, Valentin Lacombe, Jose Manuel Mascaro, Juan I. Arostegui, Yun Zhang, Xuejun Zeng

**Affiliations:** 1 Department of Family Medicine & Division of General Internal Medicine, Department of Internal Medicine, Peking Union Medical College Hospital, State Key Laboratory of Complex Severe and Rare Diseases, Chinese Academy of Medical Sciences, Peking Union Medical College Hospital, No. 1 Shuaifuyuan, Dongcheng District, Beijing, 100730 China; 2 Department of Rare Diseases, State Key Laboratory of Complex Severe and Rare Diseases, Department of Rheumatology and Clinical Immunology, National Clinical Research Center for Dermatologic and Immunologic Diseases (NCRC-DID), Key Laboratory of Rheumatology and Clinical Immunology, Peking Union Medical College Hospital (PUMCH), Ministry of Science & Technology, Ministry of Education, Chinese Academy of Medical Sciences & Peking Union Medical College, Beijing, China; 3 Department of Hematology, Peking Union Medical College Hospital, Chinese Academy of Medical Sciences, Beijing, China; 4 Médecine interne et polyvalente, CH du Haut-Anjou, Château-Gontier, France; 5 Médecine interne et immunologie clinique, CHU d’Angers, Angers, France; 6 Department of Dermatology, Hospital Clínic, Barcelona, Spain; 7 Institut d’Investigacions Biomèdiques August Pi i Sunyer (IDIBAPS), Barcelona, Spain; 8 University of Barcelona, Barcelona, Spain; 9 Department of Immunology, Hospital Clínic, Barcelona, Spain

**Keywords:** VEXAS Syndrome, *UBA1* Gene Variant, Clinical Characteristics, Screening tool

## Abstract

**Background:**

VEXAS syndrome is a severe autoinflammatory disease characterized by systemic inflammation, rheumatic manifestations, and hematologic abnormalities. Its clinical heterogeneity and overlap with other conditions complicate diagnosis. No case series have been reported from China. This study aimed to (1) describe the clinical features of Chinese patients with VEXAS syndrome and (2) propose screening recommendations to facilitate earlier identification.

**Methods:**

We conducted a retrospective cohort study at Peking Union Medical College Hospital, enrolling 57 male patients with unexplained systemic inflammation and hematologic abnormalities. *UBA1* gene sequencing was performed using bone marrow or peripheral blood samples. Clinical and laboratory data were extracted from electronic medical records. A screening model was developed using LASSO regression based on core features of confirmed cases and externally validated in two independent cohorts undergoing *UBA1* sequencing.

**Results:**

Among 57 suspected cases, 21 carried pathogenic *UBA1* variants. Compared with non-VEXAS patients, VEXAS patients more frequently showed older age at onset, skin lesions, chondritis, macrocytic anemia, and characteristic bone marrow vacuolization. Importantly, skin lesions were the initial presenting symptom in over half of VEXAS patients, whereas fever predominated in the non-VEXAS group. Rare manifestations included glomerulonephritis, acute cerebral infarction, and pulmonary arterial involvement. A novel *UBA1* variant (p.Thr318Met) was also identified. A screening model incorporating age at onset, skin lesions, chondritis, macrocytic anemia, and bone marrow vacuoles demonstrated good sensitivity and specificity in an external validation cohort.

**Conclusion:**

This study is the first to characterize the clinical features of VEXAS syndrome in a Chinese population and to delineate its phenotypic spectrum. The proposed screening tool provides a practical aid for clinicians in identifying suspected cases and may facilitate improved diagnostic pathways and patient outcomes.

**Supplementary Information:**

The online version contains supplementary material available at 10.1186/s13023-026-04380-9.

## Introduction

VEXAS syndrome (vacuoles, E1 enzyme, X-linked, autoinflammatory, somatic), first identified in 2020 and is a rare and recently recognized autoinflammatory disease characterized by somatic variants in the *UBA1* gene, primarily affecting adult males [1, 2]. The hallmark features of the syndrome include systemic inflammation, cytopenia, and vacuolation in hematopoietic precursor cells [3]. Despite increasing awareness of VEXAS syndrome, diagnosis remains challenging due to overlapping clinical manifestations with other inflammatory and hematological diseases [4, 5]. The rarity and highly variable presentation of the syndrome further contribute to delayed diagnoses or misdiagnoses.

To date, most published studies on VEXAS syndrome have been case series or individual case reports [6–10]. While these studies have played an important role in increasing disease awareness and outlining the initial clinical spectrum, they are limited in their ability to identify disease-specific risk factors or to distinguish VEXAS from phenotypically similar conditions. In contrast, case-control studies allow for systematic comparisons and are more effective in identifying discriminative clinical and laboratory markers, facilitating the development of screening tools and improving the accuracy of early diagnosis, thereby providing higher levels of evidence.In addition, no case series of VEXAS syndrome have been reported from China. Given the country’s large population and distinct genetic background, a systematic clinical investigation of VEXAS syndrome in the Chinese population is of great importance. This study represents the first case-control study of VEXAS syndrome in China. Using a phenotype-first approach, we enrolled 57 patients with persistently elevated inflammatory markers and hematologic abnormalities. Following multidisciplinary team (MDT) evaluations by rheumatologists, hematologists, and general internists, 21 patients were ultimately confirmed to have VEXAS syndrome, with *UBA1* variants verified by genetic sequencing.

This study provides detailed descriptions of the clinical manifestations, treatment responses, and prognoses of these patients. Furthermore, we proposed a screening tool based on clinical and laboratory findings to aid in the timely diagnosis of VEXAS syndrome, particularly in settings where genetic testing is less accessible.

## Methods

### Study population and design

Beginning in January 2020, patients treated at the outpatient or inpatient departments of Peking union medical college hospital (PUMCH) were enrolled in this study if they met the following inclusion criteria:

1. Recurrent fever and/or persistent systemic inflammation, with C-reactive protein (CRP) levels consistently exceeding 20 mg/L;
2. Presence of at least one hematologic abnormality: anemia (hemoglobin < 120 g/L), thrombocytopenia (platelet count < 100×10⁹/L), or leukopenia (white blood cell count < 4×10⁹/L);
3. Absence of a definitive diagnosis after consultation with rheumatology or hematology specialists, or following MDT discussion. In the absence of evidence for infection or malignancy, VEXAS syndrome was considered as a possible inferential diagnosis;
4. Underwent myeloid-malignancy targeted next-generation sequencing (NGS) including *UBA1*, whole exome sequencing(WES) or whole genome sequencing (WGS).

A total of 57 patients met these criteria and were enrolled, of whom 21 were confirmed to carry *UBA1* pathogenic variants and 36 were *UBA1*-wild type. Clinical data were extracted from the hospital’s electronic medical record system. The study was conducted in accordance with the Declaration of Helsinki and was approved by the Ethics Committee of Peking Union Medical College Hospital (I-25PJ1435). The requirement for written informed consent was waived due to the retrospective nature of the study, minimal risk to participants, and the fact that some patients were deceased or lost to follow-up. No identifiable personal information was disclosed.

### Assessment of bone marrow vacuolization

Hematopathologists evaluated cytoplasmic vacuolization in granulocytic and erythroid precursor cells by reviewing 10–15 bone marrow smears per patient using digital images stored in the electronic medical record system. Based on the frequency and distribution of vacuoles, cases were classified into three categories: none, low, or high. “None” was defined as no evident cytoplasmic vacuoles observed across the assessed slides. “Low” indicated a small number of vacuoles (typically 1–2 per precursor cell, and in a minority of cells), with vacuolated cells appearing in fewer than one-third of the slides. “High” referred to a significantly increased number of vacuoles (≥ 3 per precursor cell, and in a majority of cells) and/or vacuolated cells present in one-third or more of the slides.

### Statistical analysis

Continuous variables were summarized as medians and ranges, while categorical variables were presented as counts and percentages. The Wilcoxon rank-sum test was used to compare continuous variables between groups, and either the chi-squared test or Fisher’s exact test was applied for categorical variables. Variables with statistically significant differences (*p* < 0.05) in univariate analysis were included in a Least Absolute Shrinkage and Selection Operator (LASSO) regression model for feature selection and screening model development.

Variables with non-zero coefficients were retained in the fitted model. The smallest non-zero coefficient was used as the reference unit, and all other coefficients were divided by this value and rounded to the nearest integer to assign weighted scores. Each patient’s total score was calculated by summing the scores corresponding to their clinical features. The diagnostic performance of the model was evaluated using a receiver operating characteristic (ROC) curve, and the optimal cutoff point was determined by maximizing the Youden index. All statistical analyses were performed using R software (version 4.0.2), with a two-sided *p*-value < 0.05 considered statistically significant.

### External validation

To evaluate the external validity of the screening model, it was applied to published data from an independent retrospective cohort in the Angers University Hospital, France, for validation [11]. 53 patients suspected of VEXAS syndrome underwent *UBA1* testing, with 12 confirmed to have *UBA1* variants. The screening model’s sensitivity and specificity were evaluated against genetic test results. In addition, a published Spanish VEXAS cohort comprising 30 patients was used for sensitivity validation [6].

## Results

Among the 57 patients clinically suspected of having VEXAS syndrome (all male), 21 were found to carry *UBA1* pathogenic variants. Their clinical characteristics are summarized in Table 1.

### Core characteristics

**Fig. 1:**
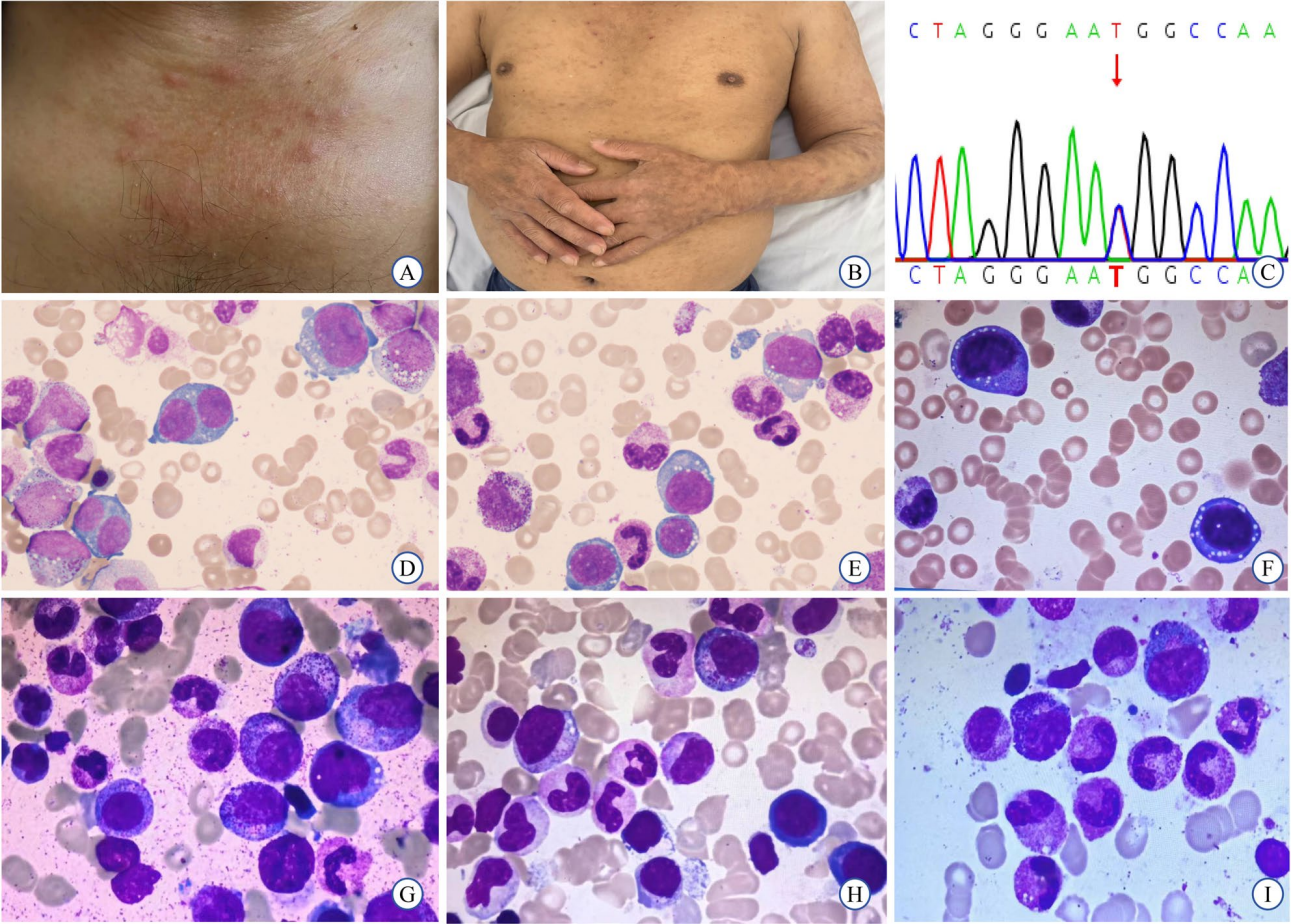
Clinical, genetic, and morphological features of VEXAS syndrome patients With *UBA1* Variants. (**A**) Cutaneous manifestation of a patient with VEXAS syndrome. (**B**) Post-inflammatory hyperpigmentation following rash resolution. (**C**) Sanger sequencing chromatogram showing *UBA1*(NM_003334.4):c.122T > C (p.Met41Thr) variant. (**D**–**F**) Cytoplasmic vacuoles in a high proportion of granulocytic and erythroid precursors in the *UBA1*-mutated group. (**G**–**I**) Low proportion of cytoplasmic vacuoles in granulocytic and erythroid precursors in the *UBA1* wild-type group

Levels of inflammatory markers (CRP and ESR) did not differ between VEXAS and non-VEXAS patients, indicating a comparable inflammatory burden across the two groups. In contrast, older age at onset, skin lesions, chondritis, ocular inflammation, macrocytic anemia, and vacuoles in marrow precursor cells were more frequently observed in the VEXAS group (*p* < 0.05). Skin manifestations in VEXAS syndrome were diverse, with histopathological findings including erythema nodosum, panniculitis, interstitial granulomatous dermatitis, neutrophilic dermatoses, and various nonspecific inflammatory changes (Fig. 1A-B). More importantly, we found that patients in the VEXAS group typically presented with rash as the initial symptom, whereas patients in the non-VEXAS group generally presented with fever as the initial symptom(*P* < 0.05). Chondritis predominantly involved the nasal and auricular regions, with no evidence of tracheal involvement. Ocular inflammation, often presenting as scleritis or uveitis, frequently occurred with chondritis. Macrocytic anemia and cytoplasmic vacuoles in myeloid and erythroid precursor cells were observed in the majority of VEXAS patients. In VEXAS patients, when vacuoles were observed on bone marrow smears, the number of vacuoles per cell typically exceeded two, with some cases showing vacuoles in both myeloid and erythroid precursors, which were features with higher specificity [12] (Fig. 1D-F). In contrast, vacuoles in the non-VEXAS group, if present, were usually limited to myeloid precursors, fewer in number (typically 1–2 per cell), and rarely seen in erythroid precursors (Fig. 1G-I). More detailed patient-level information is provided in Tables S1 and S2.

### Rare manifestations

In addition to core characteristics, some patients exhibited clinical features which were rarely reported in the literature. One patient developed symptoms at age 27, second only to the 23-year-old case reported by Sánchez-Hernández et al., challenging the widely held belief that VEXAS syndrome predominantly presents in individuals aged 50 and above [13, 14]. Two patients developed glomerulonephritis, presenting with hematuria, proteinuria, and normal creatinine levels. Base on the exclusion of other causes, the renal damage was thought to be related to VEXAS syndrome. Unfortunately, a kidney biopsy was not able to be performed, so the exact pathological type remains unknown. In one of these cases, urinalysis normalized following corticosteroid treatment. Although prior studies have reported renal involvement in VEXAS, including acute kidney injury and acute tubulointerstitial nephritis [15, 16]. Our findings suggest a potential new subtype of renal involvement in VEXAS. Three patients developed peripheral neuropathy. One patient had neuropathy at disease onset, while the other two developed symptoms approximately one year after the onset, confirmed by electromyography. Intriguingly, two patients suffered an acute cerebral infarction despite lacking common metabolic risk factors, marking another rare neurological manifestation of VEXAS that had not yet been reported. A single patient exhibited distal pulmonary arterial involvement, characterized by a beaded appearance.

### Variant characteristics

In this study, three types of *UBA1* variants—p.Met41Thr; p.Met41Leu; and p.Met41Val—were observed with roughly similar frequencies (Supplementary Table S3, Figure S1). The overall distribution of clinical syndromes was comparable across these variant types(*p* > 0.05). However, patients with the p.Met41Val variant were more likely to exhibit neutropenia and thrombocytopenia. The prevalence of neutropenia was 83.3% in the p.Met41Val group, 57.1% in the p.Met41Leu group, and 28.6% in the p.Met41Thr group (*p* = 0.124). Thrombocytopenia was observed in 50% of p.Met41Val carriers, 33.3% of p.Met41Leu carriers, and was absent in those with the p.Met41Thr variant (*p* = 0.055). In addition, one patient harbored two concurrent *UBA1* variants, including p.Met41Val (VAF 88.8%) and a splice-site mutation, c.1181G > C (VAF 3.8%). We also identified a novel *UBA1* variant, p.Thr318Met, located in exon 10 (NM_003334: c.953 C > T). The patient carrying this variant exhibited clinical features highly consistent with those observed in patients with canonical *UBA1* mutations, including persistent high-grade systemic inflammation, skin rash, macrocytic anemia, vacuolated bone marrow precursor cells, and glucocorticoid dependence.

### Treatment and prognosis

According to the FRENVEX remission criteria [17], among 18 patients, 3 (16.7%) achieved complete remission (CR), 6 (33.3%) achieved partial remission (PR), and 9 (50.0%) were classified as non-responders (NR) (more detailed information is provided in Table S4). Treatment responses were highly heterogeneous. Among the three patients who achieved CR, one responded to glucocorticoids combined with mycophenolate mofetil (MMF), subsequently discontinued glucocorticoids, and maintained remission on MMF monotherapy. One patient who was refractory to glucocorticoids plus cyclosporine A achieved CR after switching to tocilizumab, while the remaining patient attained CR after three cycles of azacitidine. Among the six patients with PR, three showed partial improvement with glucocorticoids with or without conventional synthetic disease-modifying antirheumatic drugs(csDMARDs). The remaining patients achieved PR following treatment modification, including switching to a JAK inhibitor after failure of glucocorticoids plus csDMARDs (*n* = 1), switching to tocilizumab after glucocorticoid failure (*n* = 1), and treatment with azacitidine after failure of sequential therapies with glucocorticoids, tocilizumab, and a JAK inhibitor (*n* = 1). During treatment, ten patients developed severe pulmonary infections requiring hospitalization. Identified pathogens included Pneumocystis jirovecii (PCP) in three cases, Legionella species in four cases, and one case of PCP complicated by Aspergillus niger infection. One patient unfortunately died from infection-related complications.

### Construction of the screening model

The diagnosis suspicion of VEXAS syndrome cannot be based on a single clinical feature but requires the combination of a combination of several characteristic manifestations. In univariate analysis, age at onset, skin lesions, chondritis, macrocytic anemia, ocular inflammation, and cytoplasmic vacuoles in myeloid and erythroid precursor cells in the bone marrow were all statistically associated with VEXAS syndrome. Since ocular inflammation was excluded from the modeling process due to its frequent co-occurrence with chondritis and its limited independent diagnostic value.

The final variables selected by the LASSO regression model comprised age at onset, skin lesions, chondritis, macrocytic anemia, and vacuoles in bone marrow precursor cells. Age at onset was dichotomized using 55 years as the optimal cutoff for VEXAS syndrome classification. Skin lesions were categorized based on the presence and timing of cutaneous involvement as follows: rash as the initial manifestation, rash as a non‑initial manifestation, or absence of cutaneous involvement. Regarding vacuoles in bone marrow precursor cells, they were recorded in a binary manner (present or absent) due to the practical challenges in reliably quantifying them into “low” versus “high” grades. Based on the regression coefficients of each variable, we constructed a weighted scoring system: the smallest non-zero coefficient (age at onset, β = 0.334) was used as the reference unit, and all other coefficients were divided by this value and rounded to the nearest integer. The corresponding scores assigned are shown in Table 2.

The total score for each patient is the sum of the points assigned to their clinical features. We used this total score to plot the ROC curve. The results showed that the area under the curve (AUC) of the model was 0.904. By maximizing the Youden index, the optimal diagnostic threshold was determined to be 7 points, with a sensitivity of 88.9% and specificity of 85.3%.

### External validation

This diagnostic model was retrospectively evaluated in an external validation cohort from Angers University Hospital in France. This cohort included 53 patients suspected for VEXAS syndrome, of whom 12 were genetically confirmed VEXAS patients and 41 were *UBA1* wild-type patients. Over the entire follow-up period, the model achieved a sensitivity of 100% (11/11, excluding 1 patient lost to follow-up at 18 months) and a specificity of 100% (39/39, two *UBA1*-negative patients were excluded due to unavailable bone marrow vacuole data, which precluded assessment of whether their scores reached 7) demonstrating good overall discriminative ability. However, it is worth noting that the model showed limited sensitivity for early diagnosis in this cohort. Specifically, the sensitivity was 66.6% (8/12) at 3 months after the first clinical assessment and increased to 90.9% (10/11) at 18 months before reaching 100% (11/11) over the full follow-up period. This suggests that the model’s diagnostic performance may improve over time as clinical manifestations evolve. In addition, we performed sensitivity validation using the published data from the Spanish cohort [6], and found a sensitivity of 96.4% (27/28; 2 patients were excluded because the presence of bone marrow vacuoles was not available, making it impossible to determine whether their score reached 7 points).

### Genetic characteristics of the non-VEXAS cohort

Genetic data were available for 32 of the 36 patients in the non-VEXAS cohort. Four patients had documented negative *UBA1* testing in their electronic medical records but raw sequencing reports were unavailable and therefore other variants could not be assessed. Among the remaining 32 patients with retrievable genomic data, 12 had no pathogenic variants detected by myeloid-malignancy targeted NGS (MM-NGS). At least one genetic variant was identified in the other 20 patients using MM-NGS, whole-exome sequencing, or whole-genome sequencing. The most frequently observed alterations involved *IDH* (total *n* = 7; *IDH1* *n* = 2, *IDH2* *n* = 5), *SRSF2* (*n* = 4), *MEFV* (*n* = 4) and *TET2* (*n* = 3). Additional variants were observed in genes including *SETBP1*,* TP53*,* DNMT3A*,* SF3B1*,* KMT2D*,* FANCA*,* CBL*,* ASXL1/ASXL* and others (Tables S5). From the perspective of current medical understanding, these genetic mutations do not appear to account for the entirety of their disease presentation.

## Discussion

The clinical features of VEXAS syndrome in this study closely resembled those reported internationally [6, 18], with most patients showing systemic inflammation, skin lesions, chondritis, macrocytic anemia, and bone marrow vacuolization. Cutaneous manifestations in VEXAS patients often occur early in the disease course, with skin rash being the presenting symptom in more than half of cases, whereas fever was the main complaint in the non-VEXAS group. This suggests that, in inflammatory hematologic disorders of unknown etiology, skin rash as an initial symptom may be an important clue to VEXAS and a visible marker of early systemic inflammation [19]. This study also identified certain distinct clinical features among Chinese patients. A high incidence of neutropenia was observed in our cohort (52.4%), considerably higher than the 10–20% reported in international cohorts [20], suggesting that *UBA1* mutations—particularly the p.Met41Val variant—may more profoundly impair granulopoiesis. Previous studies have shown that p.Met41Val leads to significantly reduced expression of the cytoplasmic isoform UBA1b compared to p.Met41Leu and p.Met41Thr [21], potentially disrupting proteostasis in hematopoietic cells and hindering neutrophil development. Furthermore, this study expands the clinical spectrum of VEXAS syndrome to include disease onset before age 30, glomerulonephritis, acute cerebral infarction, and pulmonary arterial involvement, providing new insights into a more comprehensive understanding of the disease. In addition, we identified a novel *UBA1* mutation. The *UBA1* p.Thr318Met variant (NM_003334.4:c.953 C > T) is a rare missense mutation located in exon 10, affecting the E1 enzymatic domain of the protein. It is extremely rare in population databases. PolyPhen-2 analysis predicted that the *UBA1* p.Thr318Met variant is probably damaging, with the highest possible score of 1.000, indicating a strong likelihood of functional impact on the protein’s E1 enzymatic activity. Further in vitro functional studies are required to confirm its biological significance.

Treatment of VEXAS syndrome remains a major clinical challenge. In this cohort, only 16.7% of patients achieved CR, while half remained non-responders, highlighting marked heterogeneity in treatment response. Glucocorticoids, with or without csDMARDs, induced PR in some patients but rarely sustained remission. Notably, one patient maintained remission on MMF monotherapy after completely discontinuing glucocorticoids, suggesting that long-term steroid-free remission may be achievable in a subset of patients, although the characteristics of such individuals require further study. With the recent adoption of biological agents and the publication of the VEXAS expert consensus [22], therapies including tocilizumab and JAK inhibitors were effective in selected patients, while azacitidine induced remission in those refractory to multiple immunosuppressive and biologic treatments, supporting the role of clonal hematopoiesis in disease pathogenesis. Treatment-related morbidity was substantial, with over half of patients experiencing severe opportunistic infections, including *Pneumocystis jirovecii* and *Legionella*. These findings underscore the need for individualized, mechanism-based treatment strategies, careful monitoring, and infection prophylaxis to optimize outcomes in this challenging disease.

Most of the current literature on VEXAS syndrome has primarily focused on clinical descriptions, and systematic diagnostic criteria remain lacking [23]. Current diagnosis mainly relies on detecting *UBA1* gene variants; however, in many resource-limited areas, the accessibility of genetic testing remains a significant barrier to diagnosis. To address this challenge, we developed an empirical clinical screening tool for VEXAS based on a cohort of patients presenting with persistent inflammatory responses accompanied by cytopenias and lacking a definitive diagnosis. We recommend that a persistent high inflammatory state serve as a key inclusion criterion for the diagnosis of VEXAS. Age at onset, skin rash, chondritis, macrocytic anemia, and bone marrow vacuolization in myeloid and erythroid precursors are relatively specific clinical manifestations. When the inclusion criteria are met and the total score reaches 7 points, the possibility of VEXAS should be considered (Table 3). Importantly, to improve the screening tool’s sensitivity for early-stage VEXAS, cytopenia was not made a mandatory inclusion criterion. Our screening score demonstrated good sensitivity and specificity in our own cohort, whereas in the external validation cohort, sensitivity declined while specificity improved significantly. Beyond population differences, this discrepancy may be attributed to the diagnostic delay commonly observed in Chinese patients, with an average interval of 23 months. Such delays may reduce the tool’s sensitivity for detecting early-stage VEXAS cases in the French cohort. Notably, the higher specificity observed in the French cohort suggests that the scoring system may be particularly effective in ruling out VEXAS during the early disease phase, thereby minimizing unnecessary genetic testing.

**Table 1:** Demographics and clinical characteristics of the cohort of patients with clinically suspected VEXAS syndrome

	*UBA1*-mutant	*UBA1*-wild-type	P-value
Male, n (%)	21 (100)	36 (100)	
Duration, median (range), months	23.0 (2-120)	18.5 (1-204)	0.810
Age, median (range), years	61.0 (30-72)	55.5 (18-74)	0.015
Age at onset, median (range), years	58.0 (27-71)	51.0 (15-73)	0.027
**Clinical symptoms**			
Initial syndrome			<0.001
Fever, n (%)	5 (23.8)	26 (72.2)	
Rash, n (%)	12 (57.1)	4 (11.1)	
Fever and rash, n (%)	1 (4.8)	1 (2.8)	
Others, n (%)	3 (14.3)	5 (13.9)	
Fever, n (%)	17 (81.0)	35 (97.2)	0.108
Rash, n (%)	20 (95.2)	16 (44.4)	<0.001
Arthritis, n (%)	9 (42.9)	9 (25.0)	0.270
Chondritis, n (%)	13 (61.9)	3 (8.3)	<0.001
Ophthalmitis, n (%)	8 (38.1)	3 (8.3)	0.016
Lung involvement, n (%)	15 (71.4)	24 (66.7)	0.938
Cardiac involvement, n (%)	1 (4.8)	5 (13.9)	0.397
PNS involvement, n (%)	3 (14.3)	1 (2.8)	0.270
CNS involvement, n (%)	2 (9.5)	1 (2.8)	0.627
Kidney involvement, n (%)	5 (23.8)	5 (13.9)	0.556
Venous thrombosis, n (%)	5 (23.8)	2 (5.6)	0.088
**Blood Examination**			
Macrocytic Anemia, n (%)	18 (85.7)	15 (41.7)	0.003
Neutropenia, n (%)	11 (52.4)	15 (41.7)	0.612
Thrombocytopenia, n (%)	6 (28.6)	14 (38.9)	0.428
ESR max, median (range), mm/h	101.0 (40-213)	100.0 (37-210)	0.205
CRP max, median (range), mg/L	100.0 (44-204)	131.5 (21-340)	0.188
ANA positive, n (%)^*^	8 (38.1)	7 (20.0)	0.243
APS positive, n (%)^#^	4 (23.5)	6 (22.2)	1.000
ANCA positive, n (%)^$^	3 (15.0)	4 (12.1)	1.000
M protein, n (%)^%^	2 (10.5)	2 (6.7)	0.636
**BM Examination**			
Pathological hematopoiesis, n (%)	8 (38.1)	14 (38.9)	1.000
Vacuoles, n (%)^&^			<0.001
None	2 (12.5)	19 (55.9)	
Low	4 (23.5)	9 (26.5)	
High	11 (68.8)	6 (17.6)	
***UBA1*** ** variant types**			
p.Met41Val, n (%)	6 (28.6)	/	
p.Met41Leu, n (%)	7 (33.3)	/	
p.Met41Thr, n (%)	7 (33.3)	/	
p.Thr318Met, n (%)	1 (4.8)	/	
C.1181G>C, n (%)	1 (4.8)	/	
**Cooccurring variants**			
* DNMT3A*, n (%)	3 (20.0)	1 (5.0)	0.292
* TET2*, n (%)	2 (13.3)	3 (15.0)	1.000
**Hematologic diagnosis**			
MGUS, n (%)^^^	2 (10.5)	2 (6.7)	0.636
MDS, n (%)	2 (9.5)	2 (5.6)	0.620
**Therapy**			
Glucocorticoid, n (%)	21 (100.0)	27 (75.0)	0.034
Immunosuppression, n (%)	14 (66.7)	18 (50.0)	0.275
Biological agents, n (%)			
IL-6 inhibition, n (%)	6 (28.6)	1 (2.8)	0.008
Jak inhibition, n (%)	6 (28.6)	1 (2.8)	0.008
Azacitidine, n (%)	3 (14.3)	0 (0)	0.045

*: ANA data were missing for 1 patients in the *UBA1* wild-type group

#: APS data were missing for 13 patients: 4 with *UBA1* variants and 9 with wild-type *UBA1*

$: ANCA data were missing for 4 patients: 1 with *UBA1* variants and 3 with wild-type *UBA1*

%: M protein data were missing for 8 patients: 2 with *UBA1* variants and 6 with wild-type *UBA1*

&: Bone marrow vacuoles refer to cytoplasmic vacuoles in myeloid and erythroid precursor cells. Vacuole data were unavailable for 6 patients: 4 with *UBA1* variants and 2 with wild-type *UBA1*

^: MGUS data were missing for 8 patients: 2 with *UBA1* variants and 6 with wild-type *UBA1*

PNS: Peripheral Nervous System; CNS: Central Nervous System; ESR: Erythrocyte Sedimentation Rate; CRP: C-reactive Protein; ANA: Antinuclear Antibody; APS: Antiphospholipid Syndrome; ANCA: Anti-Neutrophil Cytoplasmic Antibody; M protein: Monoclonal protein; BM: Bone Marrow; UBA1: Ubiquitin-like Modifier Activating Enzyme 1; Val: Valine; Leu: Leucine; Thr:Threonine; MGUS: Monoclonal Gammopathy of Undetermined Significance; MDS: Myelodysplastic Syndromes; IL-6: Interleukin-6; JAK: Janus Kinase

Maeda et al. previously proposed a scoring tool to predict the presence of *UBA1* variants, which includes five clinical parameters: age over 50 years (1 point), skin rash (1 point), pulmonary involvement (1 point), chondritis (1 point), and macrocytic anemia (2 point). A total score of 3 or more indicated a likelihood of VEXAS [23]. This tool was designed primarily for large-scale preliminary screening in outpatient settings [24], exhibiting high sensitivity but relatively low specificity (76% in the French cohort and 50% in our cohort). In contrast, our screening tool was developed in the context of VEXAS syndrome as a rare disease, which is not typically a primary diagnosis in clinical practice. It is intended to assist in identifying highly suspected VEXAS cases after exclusion of other known diagnoses or when existing diagnoses fail to fully explain the patient’s clinical presentation. Therefore, our screening tool is more suitable for complex or undiagnosed cases rather than broad screening of the general population. Given the relatively high specificity demonstrated in external validation, we further recommend that in settings with limited access to genetic testing, a provisional clinical diagnosis of VEXAS syndrome may be considered when the total score reaches 7 points. This would enable timely initiation of targeted treatment. It is important to emphasize that this tool is not intended to replace genetic sequencing, but rather to help clinicians efficiently identify patients who warrant sequencing, thereby improving diagnostic efficiency and reducing unnecessary testing burden.

Beyond the well-defined VEXAS syndrome, the etiology of many hematologic disorders accompanied by systemic inflammation remains elusive. In our control cohort, most patients lacked a definitive diagnosis. Among the 20 patients carrying at least one gene mutation, 7 patients (35%) harbored *IDH1* or *IDH2* mutations, a frequency far exceeding the background prevalence of age-related clonal hematopoiesis of indeterminate potential (CHIP). This striking enrichment suggests that *IDH* mutations may reflect an underlying hematologic inflammatory process. *IDH* mutations are neomorphic alterations that generate the oncometabolite 2-hydroxyglutarate (2-HG), which inhibits TET2 activity and induces widespread epigenetic dysregulation and impaired myeloid differentiation. Previous studies have demonstrated that *IDH* mutations are enriched in myeloid neoplasms with autoimmune or inflammatory manifestations, particularly in seronegative rheumatoid arthritis [25]. Experimental evidence also shows that *IDH*-mutant monocytes and macrophages exhibit a proinflammatory phenotype with upregulation of cytokines such as IL-18 [26–28]. Together, these data suggest that *IDH*1/2 mutations may drive distinct autoinflammatory phenotypes through a metabolically mediated inflammatory pathway. Targeted therapeutic approaches, such as mutant-IDH inhibitors, may therefore be of clinical relevance in this subset of patients. We are currently expanding our cohort and undertaking more comprehensive genomic and clinical investigations to determine whether *IDH*-associated hematologic inflammatory disease represents a distinct clinical entity with unique biological features.

### Limitations

This study has limitations. The small sample, retrospective design, and single-center setting limit generalizability. Although external validation was performed, the limited number of cases calls for multicenter studies. Our hospital serves as a national tertiary referral center, and patients are referred from both local and nationwide sources; VEXAS is likely underdiagnosed in our region due to limited clinical awareness. Nevertheless, this is the first systematic report of VEXAS in China and provides real-world screening recommendations, which may also inform international strategies.

In conclusion, this study expands understanding of VEXAS by reporting the first Chinese cohort with *UBA1* variants. The highlighting its clinical and genetic heterogeneity and emphasizing the need for novel therapeutic strategies. The proposed screening tool provides clinicians a practical framework for identifying suspected VEXAS cases, especially where genetic testing is limited. These contributions lay the foundation for early recognition and future research on this emerging disease.

**Table 2:** Scoring system based on LASSO regression for diagnosing VEXAS syndrome

	Coefficient	Categories	Point
Age at onset ≥ 55 years	0.334	No/Yes	0/1
Skin lesions	0.616	No/Non-initial/Initial	0/2/4
Chondritis	0.999	No/Yes	0/3
Macrocytic anemia	0.566	No/Yes	0/2
Vacuoles	0.706	No/Yes	0/2

**Table 3:** Screening recommendations for VEXAS syndrome

Criteria	Items		
Inclusion Criteria	Persistent high inflammatory state, primarily characterized by elevated inflammatory markers (CRP)		
	Absence of evidence supporting infections or neoplastic diseases.		
Clinical Criteria	Age at onset ≥ 55 years		1 piont
	Skin lesion		
	Absent		0 point
	Non-initial symptom		2 point
	Initial symptom		4 point
	Chondritis		3 point
	Macrocytic anemia		2 point
	Bone marrow vacuolization in granulocytic and erythroid precursors		2 point
Screening Recommendation	*UBA1* genetic testing is recommended in individuals who meet all inclusion criteria and have a total clinical score ≥ 7 points.		

## Electronic Supplementary Material

Below is the link to the electronic supplementary material.

Supplementary Material 1

## Data Availability

The datasets generated for this study are available on request to the corresponding author.

## Abbreviations

AUC: Area Under the Curve
CRP: C-Reactive Protein
csDMARDs: Conventional synthetic Disease-Modifying Antirheumatic Drugs
ESR: Erythrocyte Sedimentation Rate
IL-6: Interleukin-6
JAK: Janus Kinase
LASSO: Least Absolute Shrinkage and Selection Operator
MDS: Myelodysplastic Syndromes
MDT: Multidisciplinary Team
MGUS: Monoclonal Gammopathy of Undetermined Significance
NGS: Next-Generation Sequencing
PCP: Pneumocystis jirovecii Pneumonia
PUMCH: Peking Union Medical College Hospital
ROC: Receiver Operating Characteristic
UBA1: Ubiquitin-like Modifier Activating Enzyme 1
VEXAS: Vacuoles, E1 enzyme, X-linked, Autoinflammatory, Somatic
WGS: Whole Genome Sequencing
WES: Whole exome sequencing
