# Supplementary material for: Clinical characteristics and a screening tool for VEXAS syndrome: a case-control study from China
Source: Orphanet J Rare Dis. 2026 Jun 18;21:231. doi: 10.1186/s13023-026-04380-9 (PMC13276995; doi:10.1186/s13023-026-04380-9)
Supplement: Supplementary file 1 — Supplementary Material 1 [file 13023_2026_4380_MOESM1_ESM.docx]

| Table S1 Clinical Characteristics of 21 VEXAS Patients | | | | | | | | | | | | | | | | | | |
| --- | --- | --- | --- | --- | --- | --- | --- | --- | --- | --- | --- | --- | --- | --- | --- | --- | --- | --- |
|  | Duration | Sex | Previous diagnosis | Previous main treatment | Initial symptoms | Age at onset | Age | Fever | Rash | Chondritis | Lung involvement | Ophthalmitis | Arthritis | Cardiac involvement | PNS involvement | CNS involvement | Kidney involvement | Venous thrombosis |
| Case1 | 6 | M | Suspected lymphoma | Antibiotics | Rash | 58 | 59 | 1 | 1 | 0 | 1 | 0 | 0 | 0 | 1 | 0 | 1 | 0 |
| Case2 | 18 | M | Thrombophilia | Anticoagulation, antibiotics | Rash | 55 | 56 | 1 | 1 | 1 | 1 | 1 | 0 | 0 | 1 | 0 | 0 | 1 |
| Case3 | 16 | M | UA | GC, MTX | Arthritis | 55 | 57 | 1 | 1 | 1 | 1 | 1 | 1 | 0 | 0 | 1 | 0 | 0 |
| Case4 | 60 | M | RP | GC, MTX, cyclosporine | Rash | 60. | 66 | 1 | 1 | 1 | 1 | 1 | 1 | 0 | 0 | 0 | 0 | 1 |
| Case5 | 25 | M | RP | Antibiotics, GC, CsA, Tac | Fever | 27 | 30 | 1 | 1 | 1 | 1 | 1 | 1 | 0 | 0 | 0 | 0 | 0 |
| Case6 | 2 | M | None | Antibiotics,GC | Fever，rash | 49 | 49 | 1 | 1 | 0 | 0 | 0 | 0 | 0 | 0 | 0 | 0 | 0 |
| Case7 | 24 | M | None | NA | Leukopenia | 43 | 44 | 0 | 0 | 0 | 1 | 0 | 0 | 0 | 0 | 0 | 0 | 0 |
| Case8 | 8 | M | EN | GC, Thal | Rash | 62 | 63 | 1 | 1 | 1 | 1 | 1 | 1 | 0 | 0 | 0 | 0 | 0 |
| Case9 | 36 | M | CCUS,EN | GC,TWHF, CTX | Rash | 55 | 58 | 1 | 1 | 0 | 1 | 1 | 1 | 0 | 0 | 0 | 0 | 0 |
| Case10 | 7 | M | Suspected lymphoma | Antibiotics, GC | Fatigue | 61 | 62 | 1 | 1 | 0 | 1 | 0 | 1 | 0 | 0 | 0 | 1 | 0 |
| Case11 | 5 | M | RP | GC | Fever | 60 | 62 | 1 | 1 | 1 | 1 | 0 | 1 | 0 | 0 | 0 | 1 | 0 |
| Case12 | 24 | M | Vasculitis,HLH | GC, IS | Fever | 57 | 59 | 1 | 1 | 1 | 0 | 0 | 1 | 0 | 0 | 0 | 0 | 1 |
| Case13 | 12 | M | HLH | GC, MTX | Rash | 49 | 50 | 1 | 1 | 1 | 0 | 0 | 0 | 0 | 0 | 0 | 0 | 0 |
| Case14 | 12 | M | None | None | Fever | 71 | 71 | 1 | 1 | 1 | 1 | 1 | 0 | 0 | 0 | 0 | 0 | 0 |
| Case15 | 23 | M | HLH / TTP | GC | Rash | 64 | 68 | 1 | 1 | 1 | 1 | 0 | 0 | 0 | 1 | 1 | 1 | 0 |
| Case16 | 48 | M | None | None | Rash | 68 | 72 | 1 | 1 | 0 | 1 | 0 | 0 | 0 | 0 | 0 | 0 | 1 |
| Case17 | 2 | M | Vasculitis,panniculitis | NA | Rash | 58 | 59 | 0 | 1 | 1 | 1 | 0 | 0 | 0 | 0 | 0 | 0 | 0 |
| Case18 | 120 | M | RP | GC, LEF, MMF | Rash | 59 | 69 | 0 | 1 | 1 | 0 | 1 | 1 | 0 | 0 | 0 | 0 | 1 |
| Case19 | 24 | M | Urticarial vasculitis | GC, TWHF, HCQ | Rash | 69 | 71 | 0 | 1 | 1 | 0 | 0 | 0 | 1 | 0 | 0 | 0 | 0 |
| Case20 | 72 | M | Vasculitis | GC, CTX, MMF | Rash | 56 | 61 | 1 | 1 | 0 | 1 | 0 | 0 | 0 | 0 | 0 | 1 | 0 |
| Case21 | 48 | M | Panniculitis | GC,MTX, JAK | Fever | 57 | 61 | 1 | 1 | 0 | 0 | 0 | 0 | 0 | 0 | 0 | 0 | 0 |
| Abbreviations: UA, Undifferentiated Arthritis; RP, Relapsing Polychondritis; EN, Erythema Nodosum; CCUS, Clonal Cytopenia of Undetermined Significance; HLH, Hemophagocytic Lymphohistiocytosis; TTP, Thrombotic Thrombocytopenic Purpura; GC, Glucocorticoids; MTX, Methotrexate; CsA, Cyclosporine A; Tac, Tacrolimus; Thal, Thalidomide; TWHF, Tripterygium wilfordii Hook F; CTX, Cyclophosphamide; IS, Immunosuppressants; LEF, Leflunomide; MMF, Mycophenolate Mofetil; HCQ, Hydroxychloroquine; JAK, Janus Kinase inhibitor; PNS, Peripheral Nervous System; CNS, Central Nervous System. | | | | | | | | | | | | | | | | | | |

| Table S2 Laboratory Parameters of 21 VEXAS Patients | | | | | | | | | | | | | |
| --- | --- | --- | --- | --- | --- | --- | --- | --- | --- | --- | --- | --- | --- |
|  | Macrocytic Anemia | Neutropenia | Thrombocytopenia | ESR max(mm/h) | CRP max  (mg/L) | ANA  positive | APS positive | ANCA positive | M protein | MGUS | Pathological hematopoiesis | MDS | Vacuoles |
| Case1 | 1 | 1 | 1 | 140 | 126 | 0 | 1 | 0 | 0 | 0 | 1 | 0 | 1 |
| Case2 | 1 | 1 | 0 | 91 | 48 | 0 | 0 | 0 | 1 | 1 | 1 | 0 | 1 |
| Case3 | 1 | 1 | 1 | 110 | 68 | 0 | 0 | 0 | 0 | 0 | 0 | 0 | 1 |
| Case4 | 1 | 0 | 0 | 99 | 96 | 0 | 0 | 0 | 0 | 0 | 0 | 0 | 1 |
| Case5 | 1 | 1 | 0 | 105 | 105 | 0 | 0 | 0 | 0 | 0 | 1 | 1 | 1 |
| Case6 | 1 | 1 | 0 | 99 | 96 | 0 | 0 | 0 | 0 | 0 | 1 | 0 | 1 |
| Case7 | 1 | 1 | 0 | 40 | 92 | 0 | 0 | 0 | 0 | 0 | 0 | 0 | 0 |
| Case8 | 1 | 0 | 0 | 101 | 77.6 | 0 | 0 | 1 | 1 | 1 | 0 | 0 | 1 |
| Case9 | 1 | 0 | 0 | 102 | 157.24 | 0 | 1 | 0 | 0 | 0 | 1 | 0 | 1 |
| Case10 | 1 | 1 | 0 | 107 | 75.91 | 1 | 1 | 1 | 0 | 0 | 0 | 0 | 1 |
| Case11 | 1 | 0 | 0 | 188 | 88 | 1 | 0 | 0 | 0 | 0 | 0 | 0 | 1 |
| Case12 | 1 | 1 | 1 | 140 | 190 | 0 | 0 | 0 | 0 | 0 | 0 | 0 | NA |
| Case13 | 1 | 0 | 1 | 88 | 91 | 0 | 0 | 0 | 0 | 0 | 0 | 0 | NA |
| Case14 | 0 | 1 | 0 | 98 | 125.13 | 1 | 0 | 0 | 0 | 0 | 0 | 0 | 0 |
| Case15 | 1 | 0 | 0 | 140 | 133 | 1 | NA | 0 | 0 | 0 | 0 | 0 | NA |
| Case16 | 0 | 1 | 1 | 60 | 159.7 | 1 | NA | 0 | NA | NA | 1 | 0 | 1 |
| Case17 | 1 | 0 | 1 | 100 | 100 | 0 | NA | NA | 0 | 0 | 1 | 1 | 1 |
| Case18 | 1 | 0 | 0 | 100 | 201 | 0 | 0 | 0 | NA | NA | 1 | 0 | 1 |
| Case19 | 0 | 0 | 0 | 80 | 44 | 1 | 1 | 1 | 0 | 0 | 0 | 0 | NA |
| Case20 | 1 | 1 | 0 | 213 | 140 | 1 | NA | 0 | 0 | 0 | 0 | 0 | 1 |
| Case21 | 1 | 0 | 0 | 130 | 204 | 1 | 0 | 0 | 0 | 0 | 0 | 0 | 1 |
| Abbreviations: ESR, Erythrocyte Sedimentation Rate; CRP, C-Reactive Protein; ANA, Antinuclear Antibody; APS, Antiphospholipid Syndrome; ANCA, Antineutrophil Cytoplasmic Antibody; M protein, Monoclonal Protein; MGUS, Monoclonal Gammopathy of Undetermined Significance; MDS, Myelodysplastic Syndrome. | | | | | | | | | | | | | |

| Table S3 Genetic Testing Results in 21 VEXAS Patients | | | | | | | | | | | | | |
| --- | --- | --- | --- | --- | --- | --- | --- | --- | --- | --- | --- | --- | --- |
|  | Detection method | UBA1 mutation type | VAF | Additional genetic mutations | | | | | | | | | |
|  |  |  |  | Gene 1 | Gene 2 | Gene 3 | | Gene 4 | | Gene 5 | | Gene 6 | |
| Case1 | MM-NGS | p.M41V | 96.2 | *EZH2*(50.8) |  | |  | |  | |  | |  |
| Case2 | MM-NGS | p.M41L | 56.6 | None |  | |  | |  | |  | |  |
| Case3 | MM-NGS | p.M41L | 81.9 | None |  | |  | |  | |  | |  |
| Case4 | WES | p.M41T | NA | Unknown |  | |  | |  | |  | |  |
| Case5 | MM-NGS | p.M41V | 84.5 | None |  | |  | |  | |  | |  |
| Case6 | MM-NGS | p.M41V | 69.6 | *TET2* (3.1) |  | |  | |  | |  | |  |
| Case7 | WES | p.M41V | NA | Unknown |  | |  | |  | |  | |  |
| Case8 | WES | p.M41T | NA | Unknown |  | |  | |  | |  | |  |
| Case9 | WES | p.M41T | NA | *DNMT3A* |  | |  | |  | |  | |  |
| Case10 | UBA1 M41-PCR-Sanger | p.M41L | NA | Not performed |  | |  | |  | |  | |  |
| Case11 | MM-NGS | p.M41L | 54.9 | *DNMT3A*(29.5) |  | |  | |  | |  | |  |
| Case12 | WES | p.M41V | NA | *TET2* | *NBAS* | | *NLRP4* | | *BRCA* | | *IKZF1* | | *PTGIS* |
| Case13 | WES of the UBA1 gene | p.M41V | 88.8 | Not performed |  | |  | |  | |  | |  |
|  |  | C.1181G>C | 3.8 |  |  |  |  |  |  |  |  |  |  |
| Case14 | UBA1 M41-PCR-Sanger | p.M41T | >20% | Not performed |  | |  | |  | |  | |  |
| Case15 | WES | p.M41T | NA | *TET2* | *BACH2* | |  | |  | |  | |  |
| Case16 | WES | p.M41L | NA | *DNMT3A* | *SPEN* | | *FAT1* | | *TNRC18* | |  | |  |
| Case17 | WES | p.M41T | NA | None |  | |  | |  | |  | |  |
| Case18 | MM-NGS | p.M41L | 33.4 | *SF3BI*(25.9) |  | |  | |  | |  | |  |
| Case19 | MM-NGS | p.M41L | 44 | None |  | |  | |  | |  | |  |
| Case20 | MM-NGS | p.M41T | 51.4 | None |  | |  | |  | |  | |  |
| Case21 | MM-NGS | p.T318M | 99.85 | *IDH2*(44.87) | *SRSF2*(25.2) | | *JAK2*(7.7) | |  | |  | |  |
| None：No mutations other than in UBA1 were detected.  Unknown：The original sequencing report is unavailable; therefore, variants in genes other than UBA1 could not be assessed.  Not performed：Testing for variants in genes other than UBA1 was not conducted.  Abbreviations: MM-NGS:myeloid malignancy-targeted NGS panel;WES:Whole Exome Sequencing | | | | | | | | | | | | | |

| Table S4 Treatment Modalities, Efficacy, and Infectious Complications in 21 VEXAS Patients | | | | | | | | |  |
| --- | --- | --- | --- | --- | --- | --- | --- | --- | --- |
|  | Glucocorticoid | Immunosuppression | IL-6 inhibition | Jak inhibition | Azacitidine | Follow-up time(months） | Response | Survival Status | Infection |
| Case1 | 70 | CSA |  |  |  | 3.00 | NR | Unknow | PCP |
| Case2 | 50 | FK506 |  | Tofacitinib |  | 15.00 | PR | Alive |  |
| Case3 | 12.5 | MTX | Tocilizumab |  |  | Lost to follow up | - | Alive | LP |
| Case4 | 45 | MTX,FK506 |  |  |  | 1.00 | NR | Alive |  |
| Case5 | 60 |  | Tocilizumab | Ruxolitinib | Azacitidine | 21.00 | NR | Alive | PCP/ANP |
| Case6 | 10 | CSA |  |  | Azacitidine | 18.00 | CR | Alive |  |
| Case7 | 50 | FK506 |  |  |  | 28.00 | NR | Alive | PI (Unidentified Pathogen) |
| Case8 | 30 | MTX |  |  |  | 6.00 | PR | Dead | LP |
| Case9 | 20 | CSA | Tocilizumab |  |  | 10.00 | CR | Alive | LP,PCP |
| Case10 | 50 | FK506 |  |  |  | 1.00 | NR | Alive |  |
| Case11 | 60 | CTX,MMF |  |  |  | 10.00 | PR | Alive | PCP |
| Case12 | 17.5 |  |  |  |  | 16.00 | PR | Alive |  |
| Case13 | 20 |  | Tocilizumab | Ruxolitinib | Azacitidine | 23.00 | PR | Alive | PI (Unidentified Pathogen) |
| Case14 | 30 |  | Tocilizumab | Tofacitinib |  | 1.00 | PR | Alive |  |
| Case15 | 22.5 |  |  |  |  | 6.00 | NR | Alive | LP |
| Case16 | 20 |  |  |  |  | Lost to follow up | - | Alive |  |
| Case17 | 60 | FK506 |  |  |  | 10.00 | NR | Alive |  |
| Case18 | 10 | MMF,MTX |  | Tofacitinib |  | 5.00 | NR | Alive |  |
| Case19 | 20 | TWHF | Tocilizumab |  |  | 1.00 | PR | Alive |  |
| Case20 | 0 | MMF |  |  |  | 4.00 | CR | Alive |  |
| Case21* | 20 |  |  | Baricitinib |  | 0.00 | - | Alive | PI (Unidentified Pathogen) |
| *Case 21 has not been evaluated for treatment response due to recent diagnosis.  Abbreviations: CSA, Cyclosporine A; FK506, Tacrolimus; MTX, Methotrexate; CTX, Cyclophosphamide; MMF, Mycophenolate Mofetil; TWHF, Tripterygium wilfordii Hook F; LP,Legionella pneumonia; PCP,Pneumocystis pneumonia; PI,Pulmonary infection; ANP,Aspergillus niger pneumonia. | | | | | | | | | |

| Table S5 Genetic mutations, initial symptoms, and suspected diagnoses in the non-VEXAS group | | | | |
| --- | --- | --- | --- | --- |
|  | Gene mutations(VAF) | Detection method | Initial symptoms | Suspected diagnosis |
| Case1 | *SRSF2*(43.4)，*IDH2* (42.5) | MM-NGS | Fever | CMML |
| Case2 | *SRSF2*(16.6) | MM-NGS | Fever | AID→MDS |
| Case3 | None | MM-NGS | Low back pain | MDS |
| Case4 | *U2AF*(42.7), *SETBP1*(24.7), *TP53*(3.0) | MM-NGS | Fever | MDS |
| Case5 | None | MM-NGS | Fever | MGUS |
| Case6 | *NLRC4* | WES | Fever | CCUS→MDS+Pyoderma gangrenosum |
| Case7 | None | MM-NGS | Pancytopenia | CCUS→MDS |
| Case8 | *SRSF2*(55.5),*RUNX1* (45.9),*TET2* (44.6),*KIT* (13.8),*TP53*(9.7) | MM-NGS | Fever | FUO |
| Case9 | *FAT1* | WES | Fatigue | MDS |
| Case10 | None | WES | Rash | AID |
| Case11 | *TET2* (24.0) | MM-NGS | Fever | Lymphoma |
| Case12 | *ETV6*(37.2) | MM-NGS | Fever | AID |
|  | *MEFV* | WES |  |  |
| Case13 | *MEFV* | WES | Fever | RP |
| Case14 | *TET2* (47.1),*IDH1* (42),*ZRSR2* (36.2) | MM-NGS | Arthralgia | CCUS+IM |
| Case15 | None | MM-NGS | Fever | CTD |
| Case16 | Unknow | WES | Fever | AID |
| Case17 | *MEFV* | WES | Myalgia | AID |
| Case18 | *CTPS1* | WES | Rash | VEXAS |
| Case19 | None | WGS | Fever | AID |
| Case20 | None | WGS | Fever | AID |
| Case21 | *MEFV* | WGS | Fever | AID |
|  | *IDH2*(43.7) | MM-NGS |  |  |
| Case22 | *IRSR2* (77.1),*IDH2* (42.7), *ASXL1*(38.6) | MM-NGS | Fever | Sweet syndrome |
| Case23 | Unknow | WES | Fever | AOSD |
| Case24 | Unknow | WES | Fever |  |
| Case25 | *IDH2* (45.9),*SRSF2* (42.8) | MM-NGS | Fever | FUO |
| Case26 | None | MM-NGS | Fever | AA |
| Case27 | None | MM-NGS | Fever and Rash | FUO |
| Case28 | None | MM-NGS | Fever | FUO |
| Case29 | *FANCA* (46.6) | MM-NGS | Fever | FUO |
| Case30 | *DNMT3A*(33.1),*SF3B1*(32.8),*IDH2*(12.0),*MPL*(9.6) | MM-NGS | Fever | MDS |
| Case31 | *IDH1* (33.2) | MM-NGS | Fever | FUO |
| Case32 | None | MM-NGS | Fever | FUO |
| Case33 | *CBL*(76.7),*ASXL*(47.7),*SETBP1*(47.1) | MM-NGS | Rash | VEXAS |
| Case34 | *KMT2D*(43.1) | MM-NGS | Rash | MDS |
| Case35 | None | MM-NGS | Fever | HLH |
| Case36 | Unknow | WES | Fever | AID |
| None, no mutation detected; Unknown, original report unavailable.  Abbreviations: MM-NGS, myeloid-malignancy targeted next-generation sequencing; WES, Whole Exome Sequencing; WGS, Whole Genome Sequencing; CMML, Chronic Myelomonocytic Leukemia; AID, Autoinflammatory Disease; MDS, Myelodysplastic Syndrome; MGUS, Monoclonal Gammopathy of Undetermined Significance; CCUS, Clonal Cytopenia of Undetermined Significance; FUO, Fever of Unknown Origin; RP, Relapsing Polychondritis; IM, Inflammatory Myopathy; CTD, Connective Tissue Disease; AOSD, Adult-Onset Still’s Disease; AA, Aplastic Anemia; HLH, Hemophagocytic Lymphohistiocytosis. | | | | |


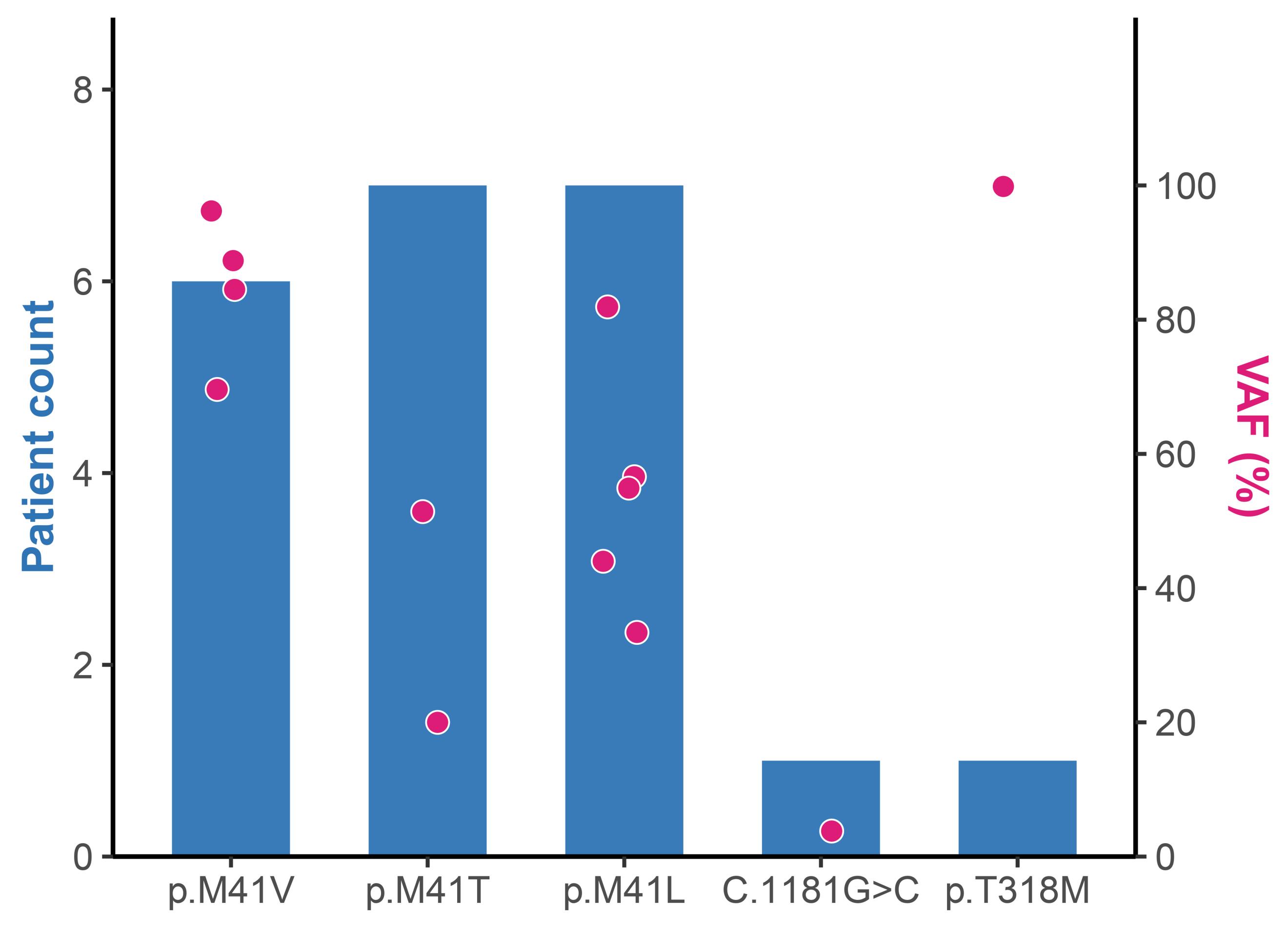


Figure S1. Patient distribution and variant allele frequencies (VAFs) of UBA1 variants. Bars indicate the number of patients carrying each variant type, and red dots represent the VAF (%) of individual patients on the secondary y-axis (0–100%).
